# Single dose denileukin diftitox does not enhance vaccine-induced T cell responses or effectively deplete Tregs in advanced melanoma: immune monitoring and clinical results of a randomized phase II trial

**DOI:** 10.1186/s40425-016-0140-2

**Published:** 2016-06-21

**Authors:** Jason J. Luke, Yuanyuan Zha, Karen Matijevich, Thomas F. Gajewski

**Affiliations:** Department of Medicine, University of Chicago, 5841 S. Maryland Ave, MC2115, Chicago, IL 60637 USA; Department of Pathology, University of Chicago, 5841 S. Maryland Ave, MC2115, Chicago, IL 60637 USA

**Keywords:** Tregs, Vaccine, Denileukin difitox, Melanoma, Immunotherapy

## Abstract

**Background:**

Depletion of CD25^+^ Tregs improves anti-tumor immunity in preclinical models. Denileukin diftitox is a recombinant fusion protein of human IL-2 and diptheria toxin fragment that also can kill CD25^+^ T cells. Prior clinical trials of denileukin diftitox suggested reduction of FoxP3^+^ Tregs and some clinical responses.

**Method:**

To investigate the immunologic effects of denileukin difitox on vaccine-specific immune responses in melanoma, a randomized clinical trial of single dose denileukin diftitox prior to vaccination versus vaccination alone in subjects with HLA-A2^+^ metastatic melanoma was performed. Treatment included randomization to a 4-peptide vaccine (Melan-A, gp100, MAGE3 and NA17 with GM-CSF emulsified in Montanide) alone or after single dose of denileukin diftitox (18 mcg/kg). Vaccine was given every 2 weeks for 3 doses and, absent clinical progression, continued every 2 weeks. Blood and tumor biopsies were obtained pretreatment and after 3 vaccinations for immunologic assessments.

**Results:**

In 17 treated subjects there were no drug-related G3-4 adverse events. One partial response and 8 stable disease were observed in 9 subjects (4 DD: 5 vaccine only) with no impact of denileukin diftitox on time to progression. Total peripheral Tregs were not significantly altered, and in 1 patient biopsy intra-tumoral FoxP3 transcripts were not reduced following denileukin diftitox. ELISA for IL2R-α demonstrated no impact on outcomes by soluble CD25 level. Immune monitoring suggested the development of modest vaccine-specific CD8^+^ T cell responses in the control group, however immunization efficacy was actually reduced in the denileukin diftitox group.

**Conclusion:**

Our results indicate that denileukin diftitox did not effectively deplete Tregs, augment T cell responses, or improve clinical activity in melanoma. Clinicaltrials.gov ID: NCT00515528; Registered August 9, 2007.

## Background

The presence of antigens expressed preferentially on tumor cells has suggested tumor-specific vaccination as an immunotherapeutic approach for the treatment of cancer. Vaccines against cancer antigens including Melan-A, gp100, MAGE, and NA17 have been associated with specific T cell immunity and occasional clinical responses [1, 2]. Various efforts have been made to improve vaccine efficacy in cancer. One approach, emulsification of granulocyte-macrophage colony-stimulating factor (GM-CSF) in Montanide, has been suggested to increase CD8^+^ T cell priming in murine models [3]. In subjects with melanoma, the administration of peptides emulsified in Montanide and GM-CSF has demonstrated significant induction of antigen-specific CD8^+^ T cells [4].

Immunologic correlates of patient response to peptide vaccines are incompletely understood, with some subjects manifesting increased peptide-specific T cell responses as detected in the peripheral blood but no regression of tumor. This general observation has suggested that mechanisms downstream from initial T cell priming may limit the effector phase of the anti-tumor immune response, thus blunting therapeutic efficacy. One mechanism of suppression of effector T cell function may be the FoxP3^+^CD4^+^CD25^+^ regulatory T cell (Treg) population. Treatment of tumor-bearing mice with anti-CD25 antibody can render mice capable of rejecting progressively growing tumors [5]. Additionally, subjects with cancer have been described to have increased Treg cells in both circulation and in the tumor microenvironment [6] with both of these associating with poor prognosis in some studies [7].

Denileukin diftitox (DAB_389_IL-2 or ONTAK®) is a recombinant fusion protein between human interleukin-2 (IL-2) and a fragment of diptheria toxin [8]. It was developed as an agent to kill T cell lymphoma cells that express the IL-2 receptor. [9] However, evidence suggests that denileukin diftitox also can kill normal T cells that express CD25. Because the majority of Tregs express high levels of CD25, it has been hypothesized that the greatest immunologic impact of denileukin diftitox may be on depleting Treg cells. A previous study of single dose denileukin diftitox followed by vaccination with tumor RNA-transfected dendritic cells noted a decrease in circulating CD4^+^CD25^+^ T cells with a greater magnitude of the specific immune response to the vaccine relative to no denileukin diftitox treatment [10]. In contrast, denileukin diftitox administered on a 5 day schedule failed to deplete CD4^+^CD25^+^ T cells from the blood of subjects with melanoma, [11] suggesting that a difference in dose or schedule could be important.

Based on the above discrepancies in the literature with denileukin diftitox, we developed a clinical protocol of administration of a single dose of denileukin diftotox prior to vaccination with a multipeptide melanoma antigen vaccine, compared to vaccine therapy alone. Herein we report on the translational biomarker analyses of this randomized phase II study.

## Methods

### Eligibility criteria

All study subjects provided voluntary written informed consent. The protocol and all amendments were approved by University of Chicago Institutional Review Board. The study was registered as ClinicalTrials.gov Identifier: NCT00515528. Vaccine preparation was performed at the University of Chicago Human Immunological Monitoring/Current Good Manufacturing Practices Facility, and denileukin diftitox was provided by Eisai.

Eligible subjects had stage IIIC or IV histologically confirmed melanoma and expression of HLA-A2, either by flow cytometry or by standard HLA typing. Study subjects could have had any number of prior treatments but could not have a history of autoimmune disease. Subjects with a history of brain metastases were eligible if these were treated and stable without corticosteroids for 4 weeks. Karnofsky performance status of ≥80 % was required as well as adequate hepatic, cardiac, renal and hematologic function defined as absolute neutrophil count ≥1500/μl, platelets ≥ 100,000/μl, and hemoglobin ≥ 10 g/dl. Lactate dehydrogenase was required to be less than 1.25 times the institutional upper limit of normal. No history of Human Immunodeficiency Virus or viral hepatitis B/C was allowed. Pretreatment biopsy was required except when deemed unsafe, and the tumor must have expressed at least two of the vaccine antigens (Melan-A, gp100, MAGE, and NA17) as determined by immunohistochemical staining of 1+ or greater for Melan-A and gp100, and/or antigen expression by RT-PCR on baseline fresh biopsies. Women of childbearing potential were required to have a negative serum pregnancy test, and subjects of both genders were required to practice adequate birth control during protocol participation.

### Clinical trial design

The clinical trial design was an open-label randomized phase II single institution study comparing administration of a four-peptide melanoma vaccine alone or after a single dose of denileukin diftitox in subjects with melanoma. Subjects were randomized 1:1 to either cohort A or B. Subjects in cohort A (vaccine alone) received vaccine immunization injected intra-dermally or subcutaneously on day 1. The vaccine was an emulsification consisting of 250 mcg each of the following peptides: Melan-A, gp100, MAGE-3, and NA17 as well as GM-CSF 125 mcg and Montanide. A second and third vaccination was given at 2 weeks and 4 weeks after the first. If there was no evidence of cancer progression, additional courses of three vaccinations administered at 2 week intervals were administered until disease progression. Subjects in cohort B received the same vaccination strategy but additionally received a single dose of denileukin diftitox (18 mcg/kg) 4 days prior to the first vaccine administration. The primary endpoints of the study were the ability of denileukin diftitox to reduce the numbers of circulating and tumor-infiltrating Tregs, as well as to increase the frequencies of vaccine-induced antigen-specific T cells. Secondary endpoints were to examine an interaction with soluble circulating CD25, toxicity assessment, and an early evaluation of clinical activity with the combination therapy.

Toxicity was graded in accordance with the Common Terminology Criteria for Adverse Events version 2.0. Dose-limiting toxicities were defined as ≥ Grade 3 in addition to Grade 2 or higher autoimmunity or visual impairment (both being criteria for study withdrawal). Dose could be delayed up to 2 weeks for toxicity prior to removal of the subject from the study. Response was evaluated every 6 weeks based on Response Evaluation In Solid Tumor (RECIST) version 1.1.

### Blood sample collection and storage

Heparinized blood was drawn before treatment, monthly during the vaccination, and at the end of the study. For patients randomized to denileukin diftitox arm, an additional blood sample was drawn 4 days after denileukin diftitox administration. All the blood samples were collected prior to a given treatment administration. PBMCs were isolated using Lymphoprep gradient centrifugation, aliquoted, and cryopreserved for later use. Serum samples were also collected before treatment and at the end of the study and stored at -80 °C until analysis.

### ELISA for soluble IL-2 receptor alpha (sIL-2R-α)

Serum sample were collected before the treatment and at the end of the study from each patient. The sIL-2R-α level was detected using the Human CD25/IL-2 R alpha Quantikine ELISA Kit (R & D Systems, Inc.)

### Flow cytometric analysis for peripheral Tregs

The fluorescence-activated cell sorting (FACS) analysis of Treg numbers were carried out using Treg detection kits from Miltenyi Biotech. Briefly, the achieved PBMCs were thawed, washed, counted, and resuspended in FACS buffer (PBS + 0.5 % BSA) at 10 x 10^6^/ml. anti-CD4 and anti-CD25 antibodies were added to 100 ml PBMCs and kept for 10 min in the dark in the refrigerator. Cells were washed and fixed in cold, freshly prepared Fixation/Permeablilization buffer for 30 min in the dark in the refrigerator. Cells were then washed and resuspended in cold Permeabilization buffer. Blocking Reagent and FoxP3 antibody were added and incubated for 30 min in the dark in the refrigerator. Cells were washed and analyzed on MACSQuant analyzer (Miltenyi Biotec). The lymphocytes were gated based on FSC and SSC parameters, CD4^+^ cells were gated in the lymphocytes gate; CD25^hi^ FoxP3^+^ cells were gated inside the CD4^+^ population. Treg numbers were calculated by multiply PBMCs numbers with % lymphocytes, % CD4^+^ cells, and % CD25^hi^ FoxP3^+^ cells.

### ELISpot for gp100, Mage-3, Melan-A and NA-17

Blood samples were collected before and every 3 cycles on treatments. PBMC were isolated and frozen for batched subsequent analysis. Direct *ex vivo* interferon (IFN)-γ Elispot analysis was performed using cryopreserved PBMC to assess for induction of antigen-specific CD8^+^ T cell responses. Briefly, one vial of frozen PBMCs from each time point was thawed and plated into anti-IFN-γ antibody (clone 1-D1K, Mabtech, Inc) pre-coated Elispot plate at 500,000 per well. Cells were then stimulated with either EBV control peptide (Invitrogen, Inc.) or one of the melanoma peptides, gp-100, Mage-3, Melan A, and NA-17 (Multiple Peptide Systems), at 50 μM for 24 h at 37 °C. Medium alone was used as negative control and P + I (PMA + Ionomycin) was used as positive control. For P + I control wells, 5000 PBMC were plated per well. After 24 h, plates were washed and incubated with a biotinylated anti-IFN-γ secondary Ab (clone 7-B6-1, Mabtech, Inc) for 2 h at room temperature, washed again and incubated with streptavidin-conjugated AP for 1 h, washed, and incubated with AP substrate. Excess substrate was removed by rinsing with tap water. Plates were then captured and counted using a CTL-ImmunoSpot S6 Core Analyzer from Cellular Technology Ltd (Cleveland, OH). All samples were analyzed in triplicate, and the mean response to the negative control was subtracted from each sample.

### qRT-PCR for FoxP3 transcripts

Ribonucleic acid (RNA) was extracted from tumor biopsy specimens using Trizol reagent (Invitrogen Inc.) according to manufacturer’s instruction. The acquired RNA samples were then used to make cDNA using High Fidelity cDNA Synthesis Kit (Roche Applied Science), followed by qRT-PCR using primer/probe sets specific for CD8 and FoxP3. β-actin was used as the internal control. Primer sequences for β-actin included: 5′-ggatgcagaaggagatcactg- 3′ and 5′ –cgatccacacggagtacttg- 3′, probe sequence for β-actin: 5′ –ccctggcacccagcacaatg- 3′. The primer sequences for CD8 included: 5′ –ccctgagcaactccatcatgt- 3′ and 5′ –gtgggcttcgctggca- 3′, the probe sequence for CD8: 5′ –tcagccacttcgtgccggtcttc- 3′. The primer sequences for FoxP3 included: 5′ – ggcactcctccaggacag- 3′ and 5′ –gctgatcatggctgggctct- 3′, the probe sequence for FoxP3: 5′ –atttcatgcaccagctct**c**aacggtgg- 3′. The qRT-PCR was run on an ABI-7300 qPCR machine (Applied Biosystem).

### Statistical analysis

Analysis of pre-treatment and post-treatment biomarker analytes was performed using paired student-t tests.

## Results

### Patient characteristics

The clinical characteristics of the 17 subjects accrued to the study are shown in Table 1. Subjects were predominately male with a median age of 63 years and median ECOG status of 1. The median number of prior therapies was two with 83 % of subjects pretreated and 29 or 24 % having had prior treatment with interleukin-2 or ipilimumab, respectively. The majority of the subjects were M stage M1C (65 %) with 41 % having 3 or more sites of disease.

**Table 1:** Subject characteristics

Total study subjects		17	
Age, median (Range), years		63	48–81
Sex	Male	14	82 %
	Female	3	18 %
ECOG PS pretreatment	0	8	47 %
	1	9	53 %
Melanoma Sub-Type	Cutaneous	15	88 %
	Mucosal	2	3 %
Prior lines of therapy, n (%)	0	3	18 %
	1	4	24 %
	2	4	24 %
	≥3	6	35 %
Median prior lines of therapy (% pretreated)		2	83 %
Prior Interleukin-2		5	29 %
Prior Ipilimumab		4	24 %
Number of metastatic sites	1	6	35 %
	2	4	24 %
	≥3	7	41 %
	M1a	1	6 %
	M1b	5	29 %
	M1c	11	65 %

### Safety and clinical activity

There were 17 subjects enrolled, with 7 (41 %) randomized to denileukin diftitox and 10 (59 %) randomized to vaccine only. Adverse reactions are listed in Table 2. All subjects completed at least three cycles of therapy. There were no grade 3 to 4 toxicities; grade 2 toxicities included nausea in 2 subjects, fatigue and anorexia in two other subjects. All other toxicities were grade 1 with the most common adverse reactions being injection site pain, headache and fatigue. All toxicities resolved spontaneously or with minimal palliative interventions.

**Table 2:** Study-agent related adverse events

Adverse Event	Grade 1	Grade 2	Grade 3–4
Injection Site Pain	10	0	0
Headache	6	0	0
Fatigue	6	1	0
Anorexia	1	1	0
Fever	2	0	0
Nausea	3	2	0
Rash	2	0	0
Infection	0	0	0
Respiratory	1	0	0
Hepatic	2	0	0
Thrombocytopenia	1	0	0
Anemia	1	0	0
Infection	1	0	0

Clinical response outcomes are listed in Table 3. One subject who received vaccine only was noted to have a RECIST response. Eight further subjects (4 receiving denileukin diftitox and 4 without) were observed to show stable disease as best response while 8 subjects demonstrated progressive disease only. No significant difference in time to progression was observed between those randomized to denileukin diftitox (4.9 months) as compared with those receiving vaccine only (4.4 months). Accrual was halted after the first 17 patients were enrolled when the immune monitoring data failed to demonstrate an improvement in any biologic endpoint with denileukin diftitox.

**Table 3:** Clinical outcomes

Response Assessment	Vaccine *N* = 10	DD + Vaccine *N* = 7	All *N* = 17
PR	1 (10 %)	0	1 (6 %)
SD	4 (40 %)	4 (57 %)	8 (47 %)
PD	5 (50 %)	3 (43 %)	8 (47 %)
Time to Progression	4.4 months	4.9 months	4.6 months

*DD* denileukin diftitox, *PD* progressive disease, *PR* partial response, *SD* stable disease

### Evaluation of circulating Treg cells with and without denileukin diftitox

**Fig. 1:**
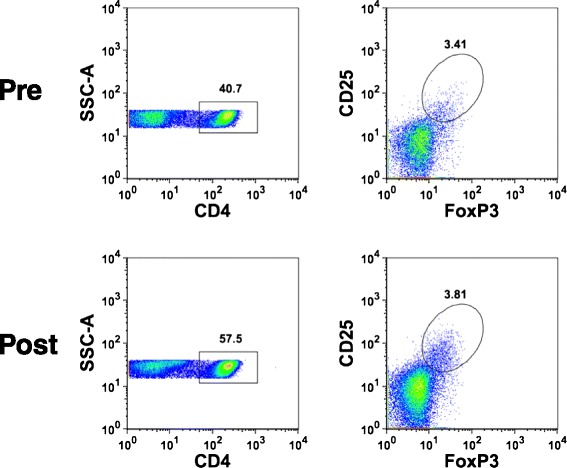
Representative flow cytometric gating for CD4^+^CD25^+^FoxP3^+^ cells. Peripheral blood mononuclear cells were isolated using density gradient centrifugation before and after patient received denileukin diftitox treatment versus no treatment. Cells were labeled with anti-CD4, anti-CD25, and anti-FoxP3 Abs from the Treg staining kit (Miltenyi Biotech, Inc.) and then analyzed using a MACSQuant analyzer

**Fig. 2:**
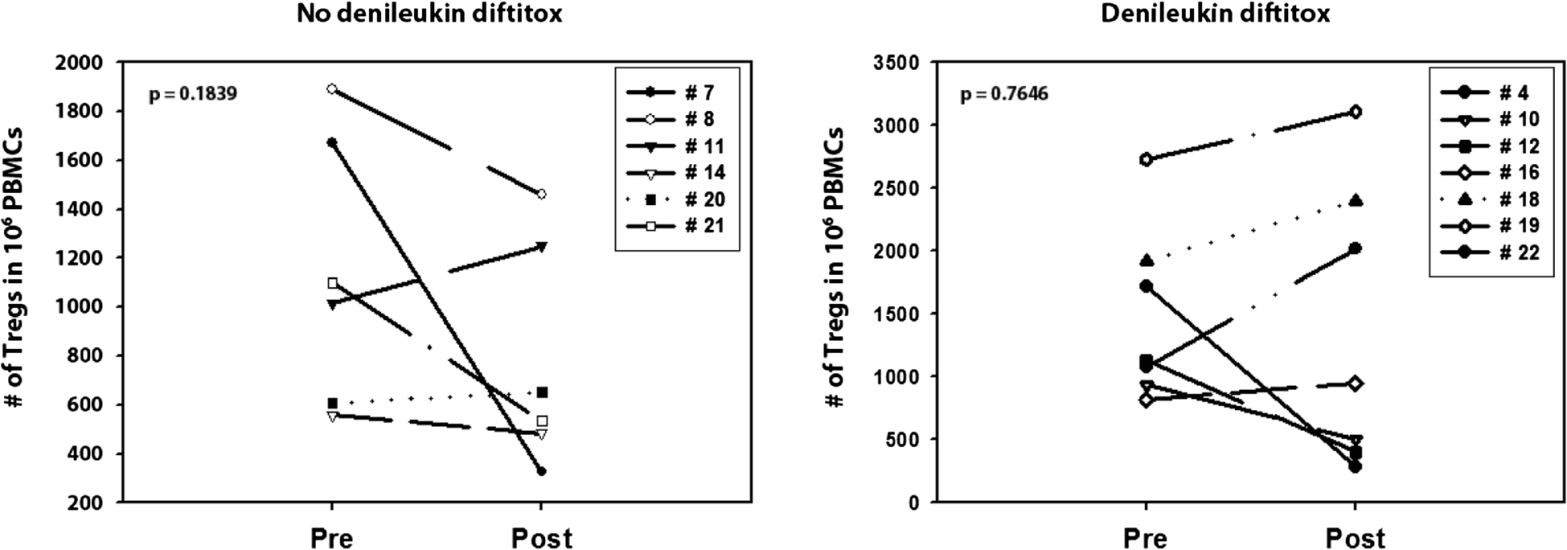
CD4^+^CD25^hi^FoxP3^+^ Treg in peripheral blood before and after denileukin diftitox treatment. Peripheral blood mononuclear cells were isolated using density gradient centrifugation before and after patient received denileukin diftitox treatment versus no treatment. In the denileukin difitox arm, peripheral blood mononuclear cells were isolated at the time of consent (pre) and before the first vaccination (post). Tregs were first labeled with anti-CD4, anti-CD25, and anti-FoxP3 Abs from the Treg staining kit (Miltenyi Biotech, Inc.) and then analyzed using a MACSQuant analyzer. Paired student *t*-test was used to determine whether there was a statistical difference between the pre- and post-treatment samples in each cohort

While some previous studies suggested that denileukin diftitox could reduce circulating Tregs, other studies did not confirm this result [10, 11]. Because dose and schedule were not identical in those trials, we chose a single dose of denileukin diftotox given prior to the initiation of vaccination. Subjects had pretreatment and on-treatment blood samples collected 4 days following denileukin diftitox (or 4 days apart without intervention for the control group). The number of circulating CD4^+^CD25^hi^FoxP3^+^ Treg cells was evaluated by flow cytometry. An example of the flow cytometry gating strategy is shown in Fig. 1. No clear population of CD25+ cells was observed outside the FoxP3 gate. As shown in Fig. 2, Treg numbers were not reproducibly reduced after a single dose of denileukin diftitox, and overall their frequencies were similar to those seen in control patients who did not receive dinileukin diftitox. These data suggest that a single dose of denileukin diftitox does not result in stable Treg cell depletion in the peripheral blood.

### Evaluation of changes in intratumoral FoxP3 transcripts

**Fig. 3:**
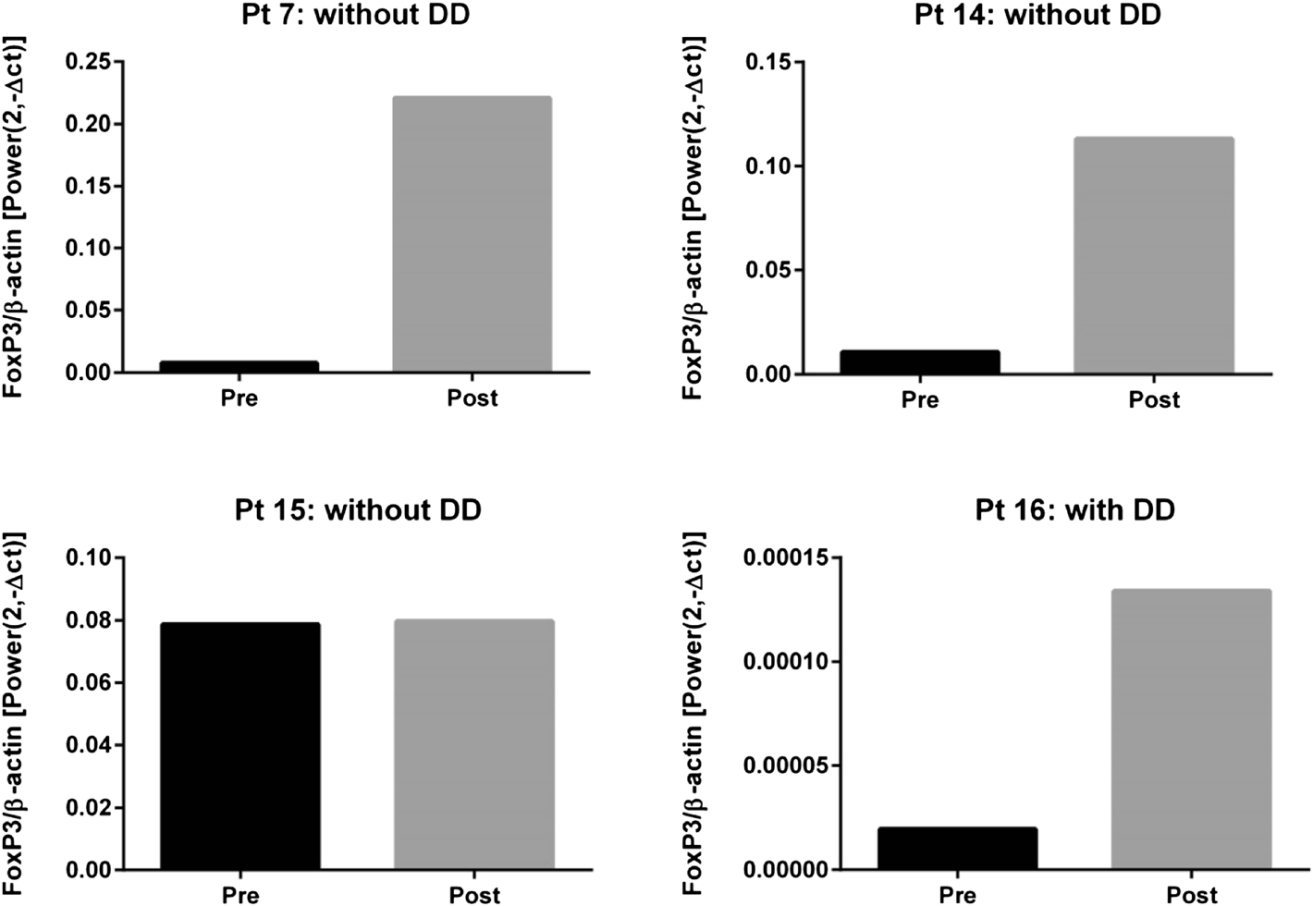
Intratumoral FoxP3 mRNA transcripts before and after treatment with and without denileukin diftitox. Fresh biopsies was collected before and after treatment when feasible and snap frozen in liquid nitrogen. RNA samples were isolated using the Trizol regent, and cDNA was synthesized using the High Fidelity cDNA Synthesis Kit (Roche Applied Science). qRT-PCR was then performed to analyze the FoxP3 transcripts levels on ABI-7300 qPCR instrument (Applied Biosystem). β-actin were used as an internal control

It was conceivable that Treg frequencies in the circulation might not be depleted by denileukin diftitox but that Tregs within the tumor microenvironment might nonetheless be significantly affected. To this end, pre- and post-treatment tumor biopsies were obtained in eligible patients to explore effects on Treg presence in the tumor microenvironment. We previously had shown that FoxP3 transcripts assessed by qRT-PCR is a quantitative assay with a broad dynamic range that reflects Treg presence as assessed by IHC [12]. Therefore, FoxP3 qRT-PCR was utilized in the current study. Paired biopsies were obtained from 4 subjects based on accessible lesions. Three of these patients (#7, 14, and 15) had been randomized to the no-denileukin diftitox group. As shown in Fig. 3, two of those patients showed increased FoxP3 transcripts in the post-treatment biopsy while one showed no change. One patient (#16) had been randomized to denileukin diftitox and showed increased FoxP3 transcripts in the post-treatment biopsy. Thus, while this is a single subject having received denileukin diftitox, we did not obtain evidence suggesting that Treg cells in the tumor microenvironment were diminished.

### Assessment of soluble circulating IL-2R-α

**Fig. 4:**
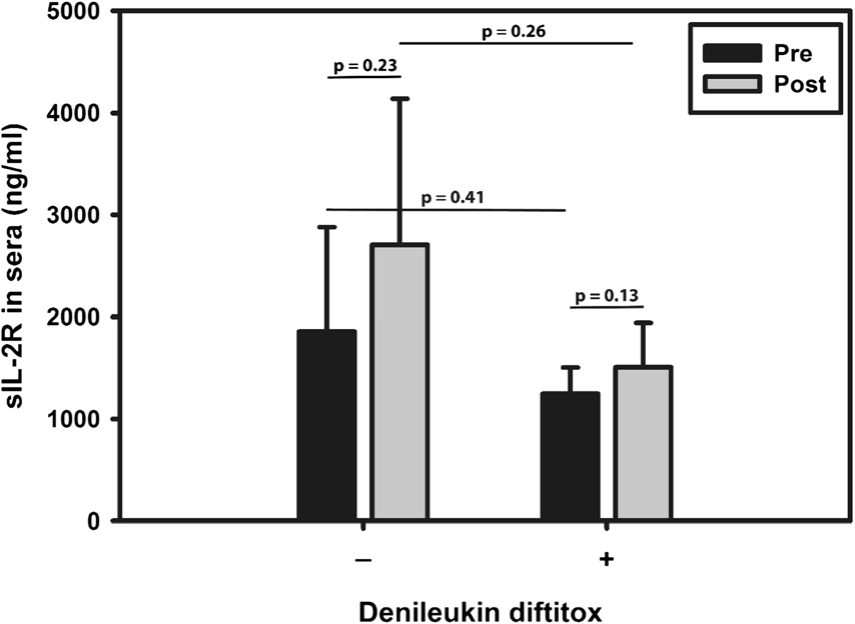
Soluble interleukin-2 receptor-α in patients treated or untreated with denileukin diftitox. Blood was drawn before and after subjects were treated on the trial. Serum was aliquoted and stored at -80 °C until analysis. Soluble interleukin-2 receptor (soluble CD25) levels were analyzed by ELISA. Student *t*-test (assuming different variance in each group) was used to determine whether there was a statistical difference between the pre and post samples in each cohort and between the two cohorts

As denileukin diftitox is a recombinant fusion protein of human IL-2 and diptheria toxin fragment, circulating IL-2R-α could potentially neutralize denileukin diftitox levels and potentially mitigate effects. Levels of IL-2R-α were therefore evaluated in peripheral circulation by ELISA assay of subjects in the study, to assess whether levels were diminished following denileukin diftitox administration. As shown in Fig. 4, no significant differences were seen in baseline levels between cohorts A and B prior (*p* = 0.41) or after treatment (*p* = 0.26). No significant differences were observed pre- or post-treatment within either cohort with (cohort B, *p* = 0.13) or without denileukin diftitox administration (cohort A, *p* = 0.23) suggesting no major interaction between circulating IL-2R-α and denileukin diftitox.

### Evaluation of peptide-specific immune activation with and without denileukin diftitox

**Fig. 5:**
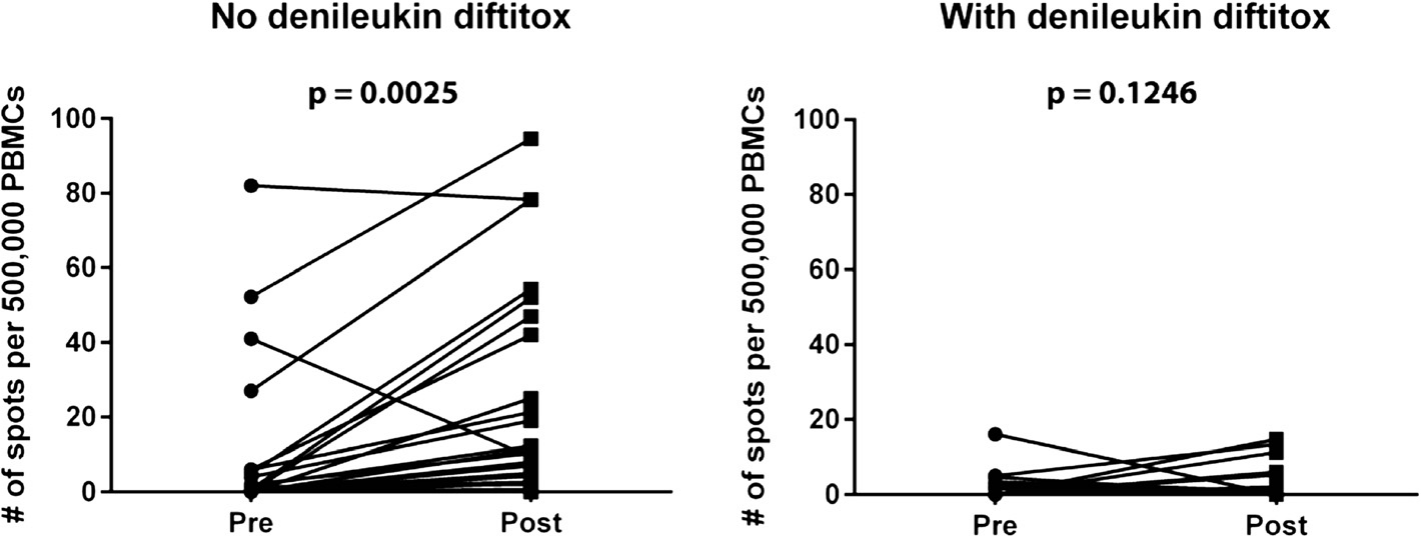
Circulating antigen-specific CD8^+^ T cells in patients treated or untreated with denileukin diftitox. Peripheral blood mononuclear cells were seeded on anti-IFN-γ Ab-coated ELISPOT plates. 5 μM of peptide was then added to each well and incubated overnight. The plates were then incubated with the biotin-labeled anti-IFN-γ Ab and developed according to manufacturer’s procedure. The plates were read and analyzed on CTL-ImmunoSpot S6 Core Analyzer from Cellular Technology Ltd. Each point represents the numerical difference of Ag-specific CD8^+^ T cells in PBMCs post-vaccine and pre-treatment for each patient. Each point represents an individual patient. Paired student *t*-test was used to determine whether there was a difference between the pre- and post- samples in each cohort

One hypothesis leading to the rationale for this combination therapy was that the administration of denileukin diftitox, through partial depletion of Tregs, might augment the induction of antigen-specific CD8^+^ T cell responses induced by vaccination. To investigate this possibility, pre- and on-treatment peripheral blood was analyzed by IFN-γ ELISPOT for the frequency of T cells specific for gp100, Mage-3, Melan-A and NA-17. As shown in Fig. 5, in the no-denileukin diftitox cohort, a modest induction of peptide-specific CD8^+^ T cells was detected following immunization in most patients, which was statistically significant for the cohort (*p* = 0.0025, paired *t*-test) and consistent with previous peptide vaccine studies [13, 14]. However, in contrast, no significant increases in specific T cell responses was observed in the denileukin diftitox cohort (*p* = 0.1246). Thus, in contrast to our initial hypothesis, these results suggest that a single dose of denileukin diftitox prior to this multipeptide melanoma vaccine failed to augment vaccine-induced T cell responses and in fact may have blunted them.

## Discussion

Recent observations have indicated the functional importance of immune inhibitory pathways in the tumor microenvironment as being rate-limiting for immune-mediated tumor control. A major subset of melanoma tumors shows evidence for a T cell-inflamed tumor microenvironment at baseline, which includes CD8^+^ T cells specific for melanoma antigens [12]. However, these same tumors show upregulation of programmed-death ligand-1 (PD-L1) and indoleamine-2,3-dioxygenase (IDO) as well as infiltration with CD4^+^FoxP3^+^ Tregs. These observations have provided a rationale for developing immunotherapy strategies that interfere with these immune suppressive pathways toward restoring T cell function at the tumor site, including monoclonal antibodies blocking PD-L1/PD-1 interactions and small molecule inhibitors of IDO. The preliminary successes of these therapies have motivated renewed attention to Tregs, and pursuit of novel therapeutics that deplete or functionally inhibit Tregs has gained critical interest. In mouse models, multiple strategies for Treg depletion have shown therapeutic effects against growing tumors in vivo. These include genetic ablation, depletion in vivo with anti-CD25 monoclonal antibodies, or in vitro depletion prior to adoptive T cell transfer [15]. Each of these manipulations can improve immune-mediated tumor control either alone or in combination with other immunotherapies [16, 17].

In the current study, the clinical and immunologic effects of a single dose of denileukin diftitox (as a strategy to target CD25-expressing cells) prior to multi-peptide vaccination were compared with multi-peptide vaccination alone in HLA-A2^+^ subjects with advanced melanoma. The treatment was well tolerated, however the addition of denileukin diftitox did not improve vaccine-induced immune responses and in fact may have inhibited them. It is unclear how denileukin diftitox might mechanistically be blunting the priming of CD8^+^ T cell responses with vaccination, but it is tempting to speculate that there may be a negative impact on CD25-expressing activated CD8^+^ T cells. It appeared that baseline T cell responses were diminished in the denileukin diftitox cohort, which could imply that pre-existing effector/memory cells might be depleted or inhibited. While this difference did not reach statistical significance, that could be limited by sample size. Beyond immune response, no clinical impact was observed, with comparable time to progression observed in the two cohorts. Correlative analysis suggested that there were no intra- or inter-cohort differences in baseline or on-treatment soluble IL-2Rα. Finally, no evidence was obtained that denileukin diftitox could deplete intratumoral Tregs. Thus, although this was a small study, we conclude that denileukin diftitox at this dose and schedule is not an effective approach for depleting Tregs and improving anti-tumor immunity in patients. Our results are somewhat at odds with the study from Dannull and colleagues, in which a single dose of denileukin diftitox was given prior to vaccination in renal cell carcinoma patients, and suggested to improve immunity [10]. However, that was a small study and non-randomized, which could explain different outcomes. In addition, vaccine formulations were different, with ours including only class I MHC epitopes. Our data are consistent with those of Attia et al. who found that denileukin diftitox given in multiple doses failed to deplete circulating CD4^+^CD25^+^ Treg cells [11]. In melanoma, a 60 patient phase II study of denileukin diftitox, at 12 μg/kg in four daily doses in every 21 day cycles, described a 16.7 % response rate with improvement in 1 year survival of subjects who experienced response [18]. While the mechanism of that apparent clinical effect is unclear, it is conceivable that there is a small subset of patients that have the potential to derive clinical benefit from this approach.

Other strategies to deplete or modulate Tregs in cancer patients are being explored and remain worthy of development. Cyclophosphamide has long been suggested to have modulating effects on Tregs, and ongoing clinical trials are evaluating cyclophosphamide in combination with anti-PD1/L1 antibody (Clinicaltrials.gov identifier NCT02383212). Conceptually similar to denileukin diftitox, studies of the recombinant anti-CD25 immunotoxins LMB-2 and RFT5-SMPT-dgA have suggested that Treg populations can be transiently depleted. However, these approaches have been limited by pharmacokinetic properties including brief half-lives and the development of neutralizing antibodies [19, 20]. Anti-CD25 monoclonal antibodies (daclizumab and basiliximab) have also been investigated in human cancer clinical trials. Administration of daclizumab in conjunction with cancer vaccine was associated with prolonged Treg depletion in peripheral blood and priming of CD8 and CD4 responses to vaccine antigens. The clinical impact of this was unclear given the small sample size and comparison to historical controls [21, 22]. Finally, monoclonal antibodies against C-C chemokine receptor type 4 (CCR4) are being investigated as an approach to deplete or modulate Tregs in patients with cancer. The anti-CCR4 antibody mogamulizumab has been shown to reduce peripheral Treg counts in patients, leading to regulatory approval for the treatment of peripheral T cell lymphomas in Japan [23]. This agent is being evaluated in conjunction with anti-PD1/L1, anti-cytotoxic T lymphocyte antigen 4 and other immunotherapies in multiple cancer histologies (Clinicaltrials.gov identifiers: NCT02476123, NCT02301130, NCT02444793).

## Conclusions

In summary, this randomized study failed to demonstrate that a single dose of denileukin diftitox could augment vaccine-specific immunity or improve clinical outcomes in subjects with advanced melanoma. Continued investigation of the hypothesis that denileukin diftitox will specifically deplete Tregs via CD25 should be discouraged, although other CD25-targeting approaches may be of interest. Broadly speaking, novel strategies for depleting or modulating Tregs in cancer patients as an immunotherapeutic strategy should remain a high priority for development.

## Abbreviations

FACS, fluorescence-activated cell sorting; a specialized technique of flow cytometry to facilitate the investigation of heterogeneous cell populations; GM-CSF, granulocyte-macrophage colony-stimulating factor; a cytokine that functions as a white blood cell growth factor; HLA, Human Leukocyte Antigen; a family of proteins that facilitate regulation of immune responses; IDO, indoleamine-2,3-dioxygenase; a heme-containing enzyme that catabolizes the amine acid L-tryptophan to N-formylkynurenine; IFN, interferon; a group of cytokine molecules that facilitate communication between immune cells; IL-2, interleukin-2; a cytokine that functions in the activation of T cells; PD-L1, programmed-death ligand-1; also known as cluster of differentiation 274, a molecule that plays a major role in suppressing immune reponses; RECIST, Response Evaluation In Solid Tumor; a set of criteria to evaluate the effectiveness of cancer treatment; Treg, regulatory T cell; a population of immune cells that maintains tolerance to self-antigens
